# ﻿New species, a new combination, and DNA barcodes of *Parachironomus* Lenz, 1921 (Diptera, Chironomidae)

**DOI:** 10.3897/zookeys.1153.98542

**Published:** 2023-03-16

**Authors:** Wen-Bin Liu, Ying Wang, Kang-Zhu Zhao, Cheng-Yan Wang, Jun-Yu Zhang, Chun-Cai Yan, Xiao-Long Lin

**Affiliations:** 1 Tianjin Key Laboratory of Conservation and Utilization of Animal Diversity, College of Life Sciences, Tianjin Normal University, Tianjin, 300387, China; 2 Shanghai Universities Key Laboratory of Marine Animal Taxonomy and Evolution, Shanghai Ocean University, Shanghai 201306, China; 3 Engineering Research Center of Environmental DNA and Ecological Water Health Assessment, Shanghai Ocean University, Shanghai 201306, China

**Keywords:** COI, new combination, new species, taxonomy, Tibetan Plateau

## Abstract

The genus *Parachironomus* has a cosmopolitan distribution including 85 valid described species worldwide. Species records and studies of the genus in the Tibetan Plateau are scarce. In this study, the genus *Parachironomus* from China is revised and two new species, *Parachironomuswangi* Liu & Lin, **sp. nov.** and *Parachironomusnankaiensis* Liu & Lin, **sp. nov.**, are described based on adult morphology and molecular data. *Paracladopelmademissum* Yan, Wang & Bu is placed in the genus *Parachironomus* as a new combination. A neighbor-joining tree was reconstructed based on all known *Parachironomus*COI DNA barcodes. A key to adult males of the genus *Parachironomus* from China is also provided.

## ﻿Introduction

The genus *Parachironomus* was erected by Lenz (1921) based on the characters of larvae and pupae with *Chironomuscryptotomus* Kieffer, 1915 as type species. Subsequently, the genus was studied by a number of authors in diﬀerent life stages and geographical areas (Edwards 1929; Townes 1945; Cranston et al. 1989; Sæther and Spies 2013). Larvae of *Parachironomus* can be found in a variety of habitats, such as standing and flowing waters, soft sediments, or within aquatic macrophytes, while others are endo- or ectoparasites on snails (Orel 2017). Among the *Harnischia* generic group, members of *Parachironomus* can separated from the similar genus *Demicryptochironomus* Lenz by the extended superior volsella with several distal setae always arising from distinct pits, and the inferior volsella with a pointed or blunt caudal projection (Yan et al. 2015). To date, 85 valid species have been reported worldwide (Lehmann 1970; Ashe and Cranston 1990; Kikuchi and Sasa 1990; Oliver et al. 1990; Sæther et al. 2000; Wang 2000; Sasa et al. 2001; Sasa and Tanaka 2001; Makarchenko et al. 2005; Spies 2008; Trivinho-Strixino et al. 2010; Yan et al. 2015; Orel 2017).

The DNA barcodes corresponding to the 658-bp fragment of the mitochondrial gene cytochrome c oxidase I (COI) has been identified as the core of a global bio-identification system at the species level (Hebert et al. 2003a, b). DNA barcodes also proved to be useful in the delimitation of non-biting midge species and has provided important evidence to confirm new species (Anderson et al. 2013; Lin et al. 2015, 2021; Giłka et al. 2018; Liu et al. 2021).

The Tibetan Plateau is located in southwest China, with a vast territory and diverse terrain. The Tibetan Plateau is one of the most important areas of biodiversity in the world because of its unique environmental and regional units, which breed unique biological communities and many unique and rare wild animals and plants. Some interesting species were discovered during the investigations of insect diversity in the Tibetan Plateau. In this paper, one new combination and two new species are proposed and described. The partial COI sequences of species in *Parachironomus* with DNA barcode analysis is conducted. A key to the known Chinese adult males of the genus is presented.

## ﻿Materials and methods

The examined specimens were caught using sweep net and light trap, stored in the dark at 4 °C, and preserved in 85% ethanol before molecular and morphological analyses. Genomic DNA was extracted from the thorax and leg using a Qiagen DNA Blood and Tissue Kit at Tianjin Normal University, Tianjin, China (**TJNU**), following the standard protocol except for the final elution volume of 100 µl. After DNA extraction, the exoskeleton of each specimen was mounted in Euparal on a microscope slide together with the corresponding antennae, legs, wing, and abdomen, following the procedures outlined by Sæther (1969). Morphological terminology follows Sæther (1980).

The color pattern of all species is described based on the specimen preserved in ethanol. Digital photographs of slide-mounted specimens were taken with a 300-dpi resolution using Nikon Eclipse 80i with Nikon Digital Sight DS-Fil camera at TJNU.

The universal primers LCO1490 and HCO2198 (Folmer et al. 1994) were adopted to amplify the standard 658-bp mitochondrial COI barcode region. Polymerase chain reaction (PCR) amplifications followed Song et al. (2018) and were conducted in a 25 µl volume including 12.5 µl 2× Es Taq MasterMix (CoWin Biotech Co., Beijing, China), 0.625 µl of each primer, 2 µl of template DNA, and 9.25 µl of deionized H_2_O. PCR products were electrophoresed in 1.0% agarose gel, purified, and sequenced in both directions at Beijing Genomics Institute Co. Ltd., Beijing, China.

Raw sequences were assembled and edited in Geneious Prime 2020 (Biomatters Ltd., Auckland, New Zealand). Alignment of the sequences was carried out using the MUSCLE (Edgar 2004) algorithm on amino acids in MEGA v. 7.0 (Kumar et al. 2016). Some published DNA barcodes of *Parachironomus* were downloaded from the Barcode of Life Data Systems (BOLD) (Ratnasingham and Hebert 2013). Before phylogenetic analysis, nucleotide substitution saturation analysis of gene sequences was performed by DAMBE version 6 (Xia 2017). The pairwise distances were calculated using the Kimura 2-Parameter (K2P) substitution model in MEGA. The neighbor-joining (NJ) tree was constructed using the K2P substitution model, 1000 bootstrap replicates, and the “pairwise deletion” option for missing data in MEGA. Novel sequences, trace-files, and metadata of the new species were uploaded to the BOLD platform.

In this study, the partial COI sequences of *Parachironomus* were submitted to online ABGD web interface (https://bioinfo.mnhn.fr/abi/public/abgd/abgdweb.html). We used the K2P nucleotide substitution model. The prior intraspecific divergence was set at between 0.001 and 0.1. The minimum relative gap width was 1.0 and other parameters were defaulted.

The holotype of two new species is deposited at the College of Fisheries and Life Science, Shanghai Ocean University (**SHOU**) and College of Life Sciences, Tianjin Normal University, Tianjin, China (**TJNU**).

## ﻿Results

### ﻿DNA barcode analysis

In this study, five COI sequences were obtained, and 19 COI sequences of *Parachironomus* were downloaded from BOLD, totaling 24 COI sequences. All sequences could be translated successfully into amino acids without indels and stop codons. MEGA analysis showed that the average total length of the sequence was 658 bp, and that there were 427 conserved sites, 231 variable sites, 196 parsimony informative sites, and 35 singleton sites. The mean nucleotide base compositions were 27.1% A, 17.9% C, 16.2% G, and 38.8% T for COI genes (Table 1). The ratio of A + T was 65.9%, which was significantly higher than that of G + C (34.1%), showing obvious AT bias, which was consistent with the bias of base composition of mitochondrial genes in most insects.

**Table 1.** The contents and nucleotide substitutions of COI gene sequences of *Parachironomus*.

	T (%)	C (%)	A (%)	G (%)	ii	si	sv	R
1^st^	48.3	8.2	40.0	3.5	144	30	44	0.7
2^nd^	25.3	18.5	27.6	28.6	205	11	1	13.3
3^rd^	42.9	27.0	13.6	16.5	216	0	0	3
Avg	38.8	17.9	27.1	16.2	565	41	44	0.9
	TT	TC	TA	TG	CT	CC	CA	CG
1^st^	76	11	18	1	10	5	2	0
2^nd^	49	5	0	0	5	35	0	0
3^rd^	93	0	0	0	0	58	0	0
Avg	218	16	18	2	14	98	2	0
	AT	AC	AA	AG	GT	GC	GA	GG
1^st^	18	2	62	5	1	0	4	2
2^nd^	0	0	59	1	0	0	1	61
3^rd^	0	0	29	0	0	0	0	36
Avg	18	2	151	6	2	0	4	98

**Figure 1.**
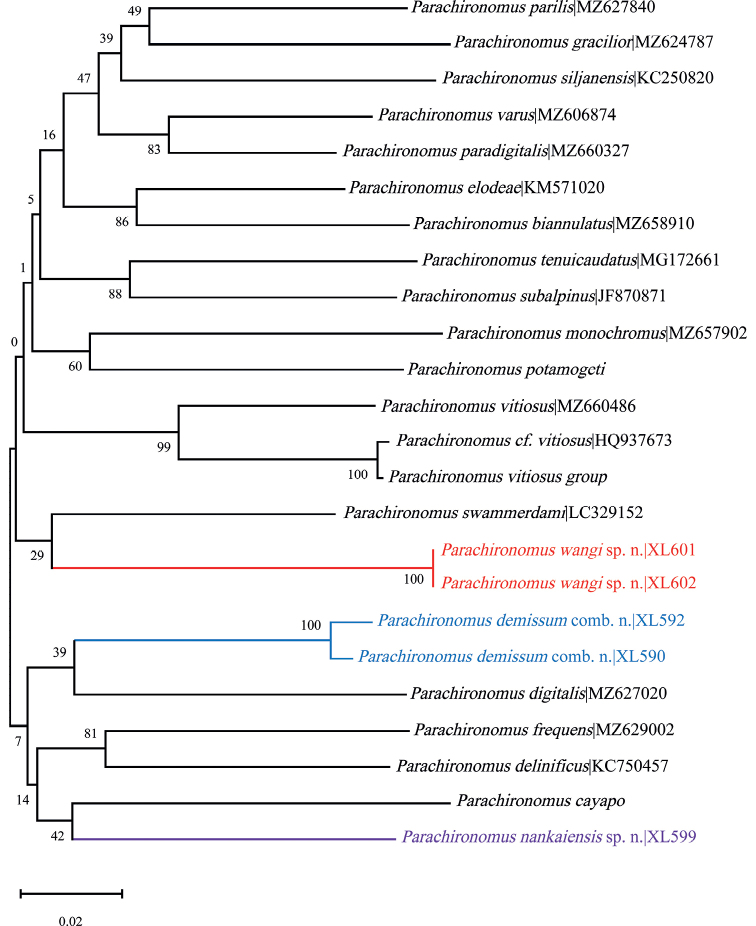
Neighbor-joining tree for 20 species of the genus *Parachironomus* based on K2P distance in DNA barcodes. Numbers on branches represent bootstrap support (> 70%) based on 1000 replicates; scale equals K2P genetic distance.

The neighbor joining tree based on available COI DNA barcodes of the *Parachironomus* revealed two species new to science (Fig. 1). *Parachironomuswangi* Liu & Lin, sp. nov. is closer to *Parachironomusbiannulatus* Staeger, 1839; and *Parachironomusnankaiensis* Liu & Lin, sp. nov. is closer to *Parachironomuscayapo* Spies, Fittkau & Reiss, 1994. The new species separate from the other sequenced species by more than 11% divergence in the COI barcode sequences (Table 2).

**Table 2.** Kimura 2-parameter pairwise genetic distances based on COI barcodes of the *Parachironomus*.

Species	Pairwise genetic distances																						
*P.tenuicaudatus*|MG172661																							
*P.varus*|MZ606874	0.13																						
*P.digitalis*|MZ627020	0.14	0.13																					
*P.frequens*|MZ629002	0.15	0.15	0.13																				
*P.parilis*|MZ627840	0.13	0.09	0.14	0.14																			
*P.gracilior*|MZ624787	0.13	0.12	0.14	0.13	0.10																		
*P.siljanensis*|KC250820	0.14	0.10	0.13	0.15	0.11	0.11																	
P.cf.vitiosus|HQ937673	0.14	0.13	0.15	0.13	0.15	0.15	0.14																
*P.monochromus*|MZ657902	0.15	0.14	0.14	0.14	0.14	0.15	0.15	0.13															
*P.vitiosus*|MZ660486	0.12	0.14	0.14	0.13	0.13	0.15	0.14	0.07	0.13														
*P.subalpinus*|JF870871	0.10	0.13	0.14	0.14	0.12	0.13	0.14	0.14	0.14	0.14													
*P.elodeae*|KM571020	0.12	0.10	0.14	0.14	0.12	0.13	0.13	0.11	0.13	0.11	0.11												
*P.vitiosus group*	0.14	0.13	0.15	0.13	0.14	0.15	0.14	0.00	0.13	0.08	0.13	0.11											
*P.potamogeti*	0.13	0.12	0.14	0.14	0.14	0.15	0.14	0.15	0.12	0.13	0.12	0.12	0.15										
*P.swammerdami*|LC329152	0.13	0.12	0.14	0.12	0.13	0.14	0.12	0.13	0.15	0.12	0.13	0.11	0.13	0.12									
*P.delinificus*|KC750457	0.14	0.13	0.13	0.11	0.14	0.13	0.14	0.13	0.14	0.13	0.14	0.13	0.13	0.14	0.14								
*P.cayapo*	0.15	0.13	0.16	0.14	0.14	0.15	0.14	0.15	0.14	0.14	0.15	0.13	0.15	0.15	0.14	0.14							
*P.paradigitalis*|MZ660327	0.12	0.07	0.13	0.14	0.10	0.13	0.11	0.11	0.13	0.13	0.13	0.09	0.11	0.13	0.10	0.12	0.12						
*P.biannulatus*|MZ658910	0.13	0.11	0.15	0.15	0.12	0.15	0.13	0.13	0.13	0.13	0.13	0.09	0.13	0.12	0.11	0.15	0.14	0.10					
*P.demissum* comb. nov. |XL592	0.14	0.14	0.11	0.13	0.14	0.13	0.14	0.13	0.15	0.13	0.13	0.13	0.12	0.13	0.13	0.12	0.14	0.13	0.15				
*P.nankaiensis* sp. nov. |XL599	0.14	0.13	0.12	0.13	0.13	0.12	0.14	0.14	0.15	0.14	0.12	0.13	0.14	0.15	0.14	0.13	0.13	0.12	0.15	0.13			
*P.demissum* comb. nov. |XL590	0.14	0.14	0.11	0.13	0.14	0.13	0.13	0.12	0.14	0.12	0.12	0.13	0.12	0.13	0.13	0.12	0.13	0.12	0.15	0.01	0.12		
*P.wangi* sp. nov. |XL601	0.14	0.14	0.14	0.15	0.14	0.15	0.16	0.14	0.15	0.14	0.14	0.14	0.14	0.15	0.12	0.14	0.16	0.14	0.15	0.12	0.14	0.12	
*P.wangi* sp. nov. |XL602	0.14	0.14	0.14	0.15	0.14	0.15	0.16	0.14	0.15	0.14	0.14	0.14	0.14	0.15	0.12	0.14	0.16	0.14	0.15	0.12	0.14	0.12	0.00

**Figure 2.**
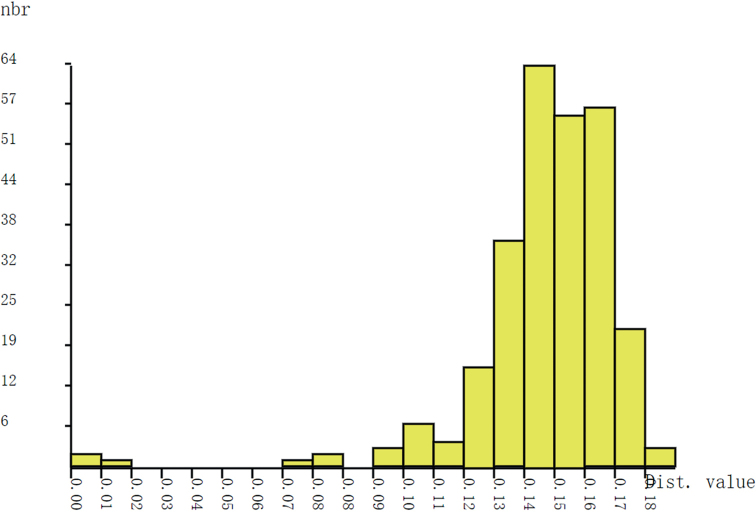
Histogram of pairwise K2P distances of 24 COI barcodes of *Parachironomus*.

When the interspecific genetic distance is greater than the intraspecific genetic distance, barcode gaps will appear through the frequency histogram of genetic data. There is an obvious barcode gap in the genetic distance of all *Parachironomus*COI sequences, which fully confirms the feasibility of COI as a DNA barcode (Fig. 2).

### ﻿Taxonomic descriptions

#### ﻿ Parachironomus demissum

Taxon classificationAnimaliaDipteraChironomidae

Yan, Wang & Bu, 2012 comb. nov.

BD3B0498-C468-54C7-B3BD-F69F044B0A39

Figs 3 , 4 , 5

Paracladopelma demissum Yan, Wang & Bu, 2012: 291.

##### Material examined.

***Holotype*.** Male (TJNU: 11430), China, Sichuan Province, Yajiang County, Sandaoqiao Town, 30.01532°N, 101.05134°E, 2460 m a.s.l., 9.VI.1996, light trap, leg: X. H. Wang. ***Paratypes*.** One male (TJNU: 11730), China, Sichuan Province, Ya’an City, Baoxing County, Xinglong Elementary School, Xihe River, 30.25800°N, 102.50387°E, 1100 m a.s.l., 19.VI.1996, light trap, leg: X. H. Wang. One male (TJNU: 12886), China, Sichuan Province, Kangding County, Wasigou River, 30.04191°N, 102.09454°E, 2000 m a.s.l., 15.VI.1996, light trap, leg: X. H. Wang. One male (TJNU: 13079), China, Sichuan Province, Shimian County, Nanyahe River, 29.11156°N, 102.23265°E, 1040 m a.s.l., 16.VI.1996, light trap, leg: X. H. Wang.

##### Additional materials.

Two males (SHOU: XL590, XL592), China, Xizang Autonomous Region, Rikaze City, Yadong County, Xiasima Town, 27.48986°N, 88.90572°E, 3032 m a.s.l., 20.VII.2014, light trap, leg: X. L. Lin.

##### Diagnostic characters.

The species can be distinguished from known species of *Parachironomus* by the following combination of characters: AR 0.58–0.67, 0.62; tergite IX with shoulder-like posterior margin; anal point triangular base, constricted in the middle, with a ridge mesally; anal tergite bands V-shaped and fused; superior volsella with a bare triangular projection apically; gonostylus slender, parallel-sided, curved medially, gradually tapered to the top.

##### Description.

**Adult males** (*n* = 6, unless otherwise stated). Total length 3.08–3.60, 3.35 mm. Wing length 1.60–1.89, 1.75 mm. Total length/wing length 1.81–2.10, 1.93. Wing length/length of profemur 2.16–2.63, 2.32.

***Coloration*.** Thorax yellowish brown with pale brown spots. Femora and tibiae of front legs yellowish green with distal parts brown, anterior 1/2 of tarsi I yellowish green, remainder of front legs dark brown; femora, tibiae and basal 1/2 of tarsi I of mid and hind legs yellowish green, remaining dark brown. Abdomen yellowish green to dark brown, with tergites I–V yellowish green, tergites VII, VIII, and hypopygium dark brown.

**Figure 3.**
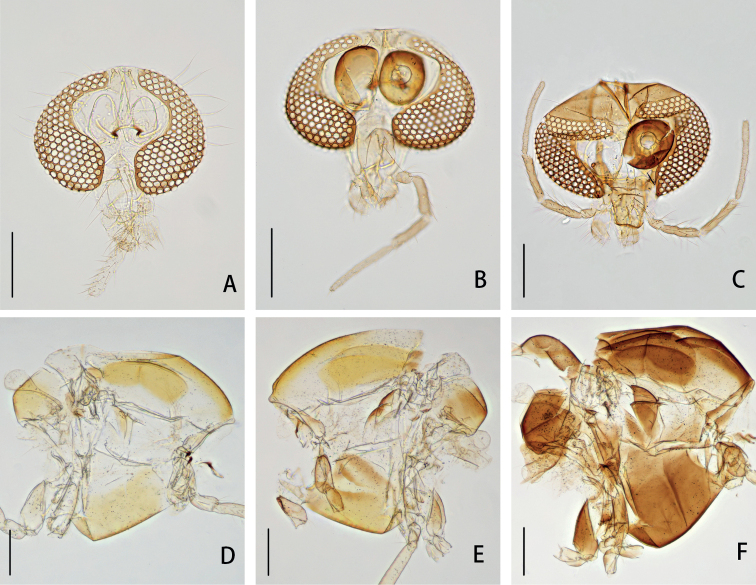
Head (**A–C**) and thorax (**D–F**) of *Parachironomus* species **A***P.demissum* (Yan, Wang & Bu), comb. nov. **B***P.wangi* Liu & Lin, sp. nov. **C***P.nankaiensis* Liu & Lin, sp. nov. **D***P.demissum* (Yan, Wang & Bu), comb. nov. **E***P.wangi* Liu & Lin, sp. nov. **F***P.nankaiensis* Liu & Lin, sp. nov. Scale bars: 200 μm.

***Head*** (Fig. 3A). Antenna with 11 flagellomeres, ultimate flagellomere 250–290, 273 (3) μm long. AR 0.58–0.67, 0.62 (3). Frontal tubercles conical, 13–25, 18 (5) μm long, 8–20, 17 (5) μm wide at base. Temporal setae 17–20, 18; including 3–4, 3 inner verticals; 5–8, 8 outer verticals; and 6–9, 8 postorbitals. Clypeus with 12–16, 15 setae. Tentorium 105–132, 118 (5) μm long, 22–28, 24 (5) μm wide. Palpomere lengths (in μm):30–42, 37 (5); 35–45, 39 (5); 95–119, 109 (5); 130–148, 139 (5); 155–220, 190 (5); Pm5/ Pm3 1.48–2.05, 1.76 (5).

***Thorax*** (Fig. 3D). Antepronotals with 2–3, 2 setae, acrostichals 5–7, 6, dorsocentrals 7–8, 8, prealars 3. Scutellum with 12–14, 13 setae.

***Wing*** (Fig. 5A). VR 1.18–1.34, 1.26. R with 13–18, 16 setae, R_1_ with 12–18, 15 setae, R_4+5_ with 12–22, 16 setae. Brachiolum with 2–3, 2 setae. Squama with 3–5, 4 setae.

***Legs*.** Front tibia with 3 subapical setae, 80–84, 82; 83–97, 91 and 85–112, 101 (5) μm long. Combs of mid tibia 37–41, 39 μm wide with 18–25, 22 μm long spur, and 41–55, 48 μm wide with 20–34, 27 μm long spur; combs of hind tibia 37–41, 39 μm wide with 23–27, 25 μm long spur, 60–66, 63 μm wide with 28–34, 32 μm long spur. Tarsomere 1 of mid and hind legs without sensilla chaetica. Lengths (in μm) and proportions of legs as in Table 3.

**Table 3.** Lengths (in μm) and proportions of legs of *Parachironomusdemissum* (Yan, Wang & Bu), comb. nov., adult male (*n* = 6).

	fe	ti	ta_1_	ta_2_	ta_3_
P_1_	632–815, 755	498–640, 586	901–1049, 996	341–434, 407	276–352, 329
P_2_	575–810, 731	524–660, 600	317–370, 349	134–180, 169	99–133, 122
P_3_	730–910, 841	698–890, 802	496–549, 521	245–290, 275	187–246, 221
	**ta_4_**	**ta_5_**	**LR**	**BV**	**SV**
P_1_	273–326, 305	152–170, 162	1.63–1.81, 1.69	1.94–1.95, 1.94	1.25–1.37, 1.34
P_2_	64–90, 84	66–70, 69	0.55–0.64, 0.58	3.75–3.90, 3.79	3.47–3.93, 3.81
P_3_	112–140, 126	80–96, 87	0.61–0.71, 0.65	3.01–3.07, 3.05	2.88–3.22, 3.15

**Figure 4.**
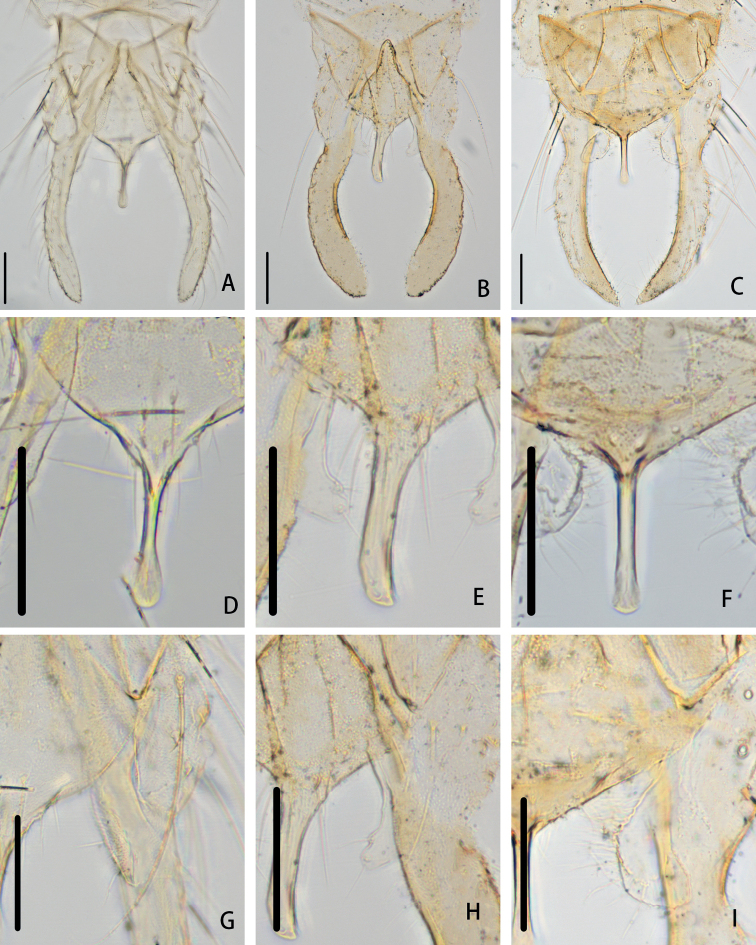
Hypopygium **A***P.demissum* (Yan, Wang & Bu), comb. nov. **B***P.wangi* Liu & Lin, sp. nov. **C***P.nankaiensis* Liu & Lin, sp. nov. Anal point **D***P.demissum* (Yan, Wang & Bu), comb. nov. **E***P.wangi* Liu & Lin, sp. nov. **F***P.nankaiensis* Liu & Lin, sp. nov. Superior volsella **G***P.demissum* (Yan, Wang & Bu), comb. Nov. **H***P.wangi* Liu & Lin, sp. nov. **I***P.nankaiensis* Liu & Lin, sp. nov. Scale bars: 50 μm.

**Figure 5.**
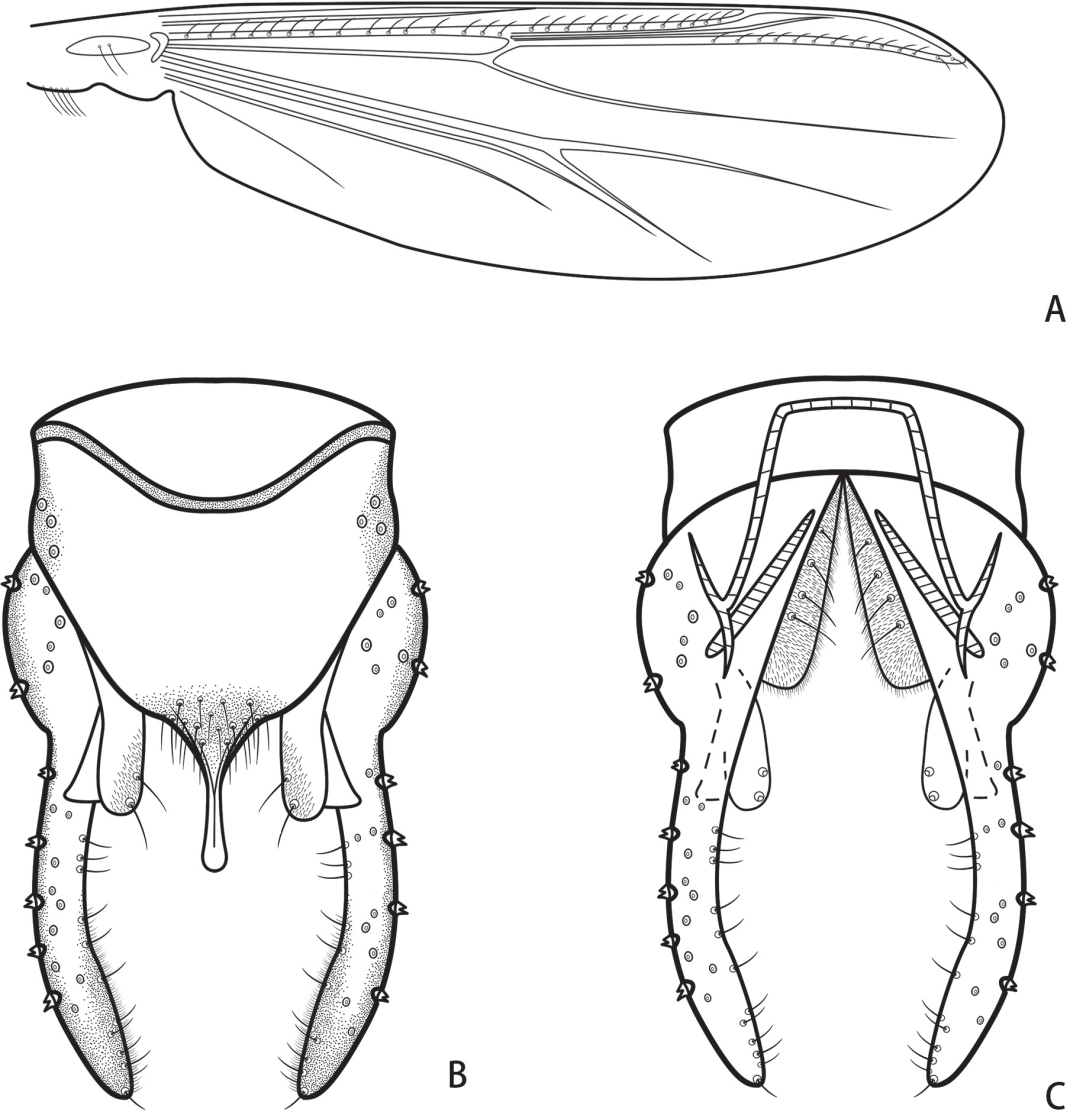
*Parachironomusdemissum* (Yan, Wang & Bu), comb. nov. holotype male **A** wing **B** hypopygium, dorsal view **C** hypopygium, ventral view.

***Hypopygium*** (Figs 4A, D, 5B, C). Tergite IX with shoulder-like posterior margin, bearing 16–24, 20 setae at base of anal point. Laterosternite IX with 2 or 3, 3 setae. Anal point 55–67, 63 μm long, 12–25, 19 μm wide at base, 10–14, 13 μm wide apically, originating from caudal margin of anal tergite, triangular base, constricted in the middle, with a ridge mesally, moderately widen to distal and apically swollen (Fig. 4D). Anal tergite bands V-shaped and fused. Phallapodeme 52–70, 61 μm long. Transverse sternapodeme 30–52, 42 μm long. Superior volsella 55–65, 61 μm long, curved medially, expanded in the apical part, apically with a bare triangular projection, bearing two subapical setae, covered with microtrichia on inner margin. Inferior volsella with moderately blunt caudal projection, not reaching beyond anal tergite margin, and covered by microtrichia (Fig. 4G). Gonocoxite 90–131, 107 μm long, with three setae on inner margin. Gonostylus 140–174, 168 μm long, slender, parallel-sided, curved medially, gradually tapered to the top, bearing 12–24, 20 setae along inner margin and one stronger seta at apex. HR 0.55–0.94, 0.68. HV 1.86–2.50, 2.09.

##### Distribution.

China (Sichuan and Xizang).

#### ﻿ Parachironomus wangi

Taxon classificationAnimaliaDipteraChironomidae

Liu & Lin sp. nov.

5C236D6B-41F2-5580-800B-721C81DBDD3D

https://zoobank.org/DE07AF99-393F-407D-826E-FCA5C9B45FE1

Figs 3 , 4 , 6

##### Material examined.

***Holotype*.** Male (SHOU: XL601), China, Xizang Autonomous Region, Shannan City, Gongga County, Gangdui Town, 29.27888°N, 90.82323°E, 3586 m a.s.l., 18.VII.2014, sweep net, leg: X.L. Lin. ***Paratypes*.** One male (TJNU: XL602), collecting data as holotype.

##### Diagnostic characters.

The species can be distinguished from known species of *Parachironomus* by the following combination of characters: frontal tubercles absent; tergite IX with triangular posterior margin; anal point slightly wider at base, parallel-sided, swollen apically; superior volsella wide, parallel-sided, bent inward at 1/3 distance from apex, apically rounded, free microtrichia; inferior volsella not reaching beyond anal tergite margin; gonostylus gradually widened distally, curved and parallel-sided, apically rounded.

##### Description.

**Adult males** (*n* = 2, unless otherwise stated). Total length 3.63–3.91, 3.77 mm. Wing length 1.85–1.91, 1.88 mm. Total length/wing length 1.96–2.05, 2.01. Wing length/length of profemur 2.74–2.79, 2.77.

***Coloration*.** Thorax yellowish brown with pale brown spots. Front legs dark brown; femora and basal 1/3 of tarsi I of mid and hind legs yellowish brown, remaining dark brown. Abdomen pale yellow to yellowish brown, with tergites I–VI pale yellow, tergites VII, VIII, and hypopygium yellowish brown.

***Head*** (Fig. 3B). Antenna with 11 flagellomeres, ultimate flagellomere 502–536, 519 μm long. AR 1.54–1.55, 1.55. Frontal tubercles absent. Temporal setae 13–14, 14; including 2–4, 3 inner verticals; 4–6, 5 outer verticals; and 5 or 6, 6 postorbitals. Clypeus with 7–9, 8 setae. Tentorium 95–128, 112 μm long, 31–34, 33 μm wide. Palpomere lengths (in μm): 26–28, 27; 37–39, 38; 92–98, 95; 106–109, 108; 176–193, 185; Pm5/ Pm3 1.91–1.97, 1.94.

***Thorax*** (Fig. 3E). Antepronotals with 1 or 2, 2 setae, acrostichals 4, dorsocentrals 8, prealars 3. Scutellum with 12 setae.

**Figure 6.**
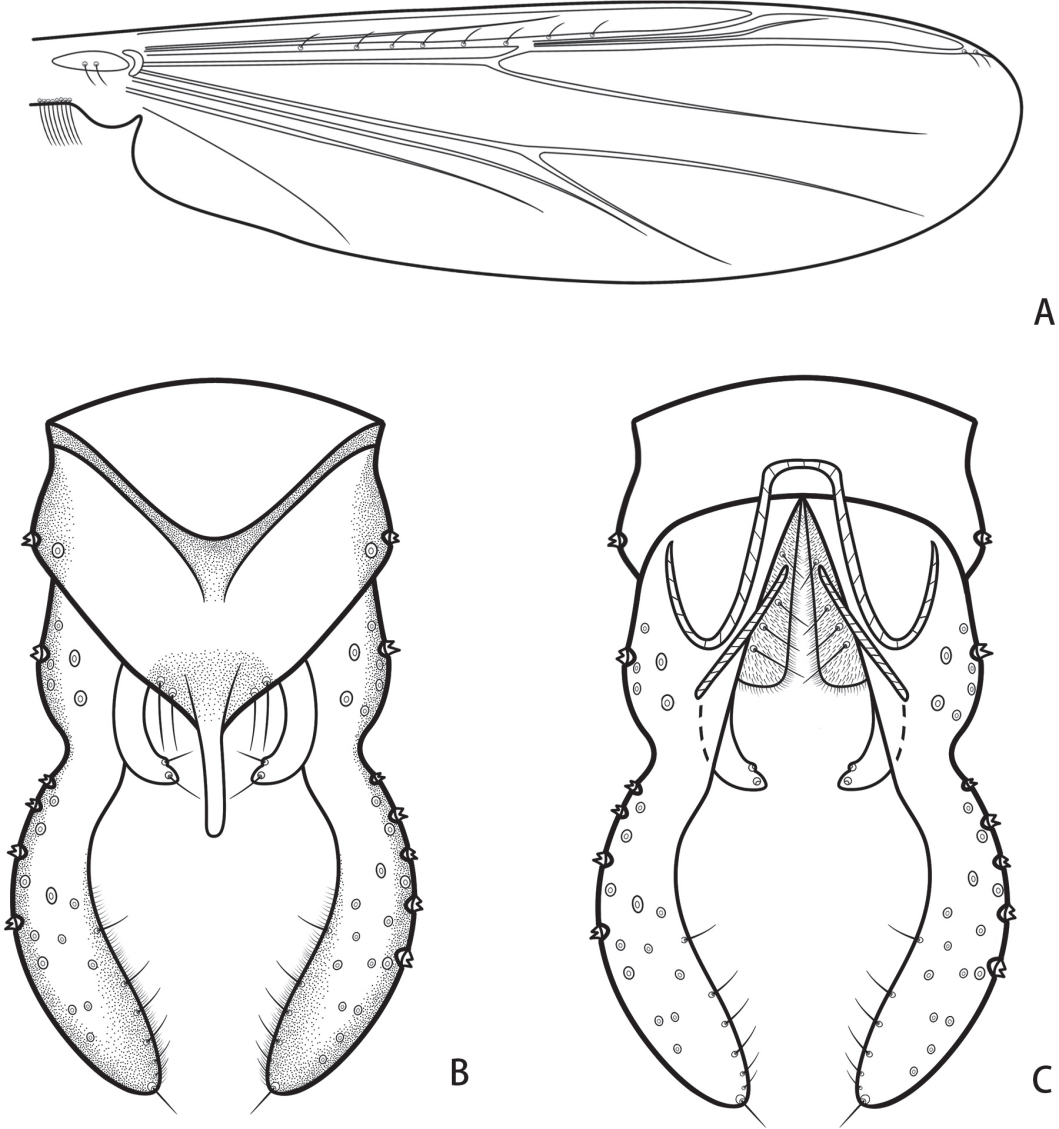
*Parachironomuswangi* Liu & Lin, sp. nov. holotype male **A** wing **B** hypopygium, dorsal view **C** hypopygium, ventral view.

***Wing*** (Fig. 6A). VR 1.10–1.16, 1.13. R with 5 or 6, 6 setae, R_1_ with 1 or 2, 2 setae, R_4+5_ with 2 setae. Brachiolum with two setae. Squama with 7–9, 8 setae.

***Legs*.** Front tibia with three subapical setae, 82–91, 87; 84 (1) and 105 (1) μm long. Combs of mid tibia 26–33, 30 μm wide with 15–25, 20 μm long spur, and 21 μm wide with 28 μm long spur; combs of hind tibia 22–26, 24 μm wide with 22–34, 28 μm long spur, 38–42, 40 μm wide with 34–38, 36 μm long spur. Tarsomere 1 of mid and hind legs without sensilla chaetica. Lengths (in μm) and proportions of legs as in Table 4.

**Table 4.** Lengths (in μm) and proportions of legs of *Parachironomuswangi* Liu & Lin, sp. nov., adult male (*n* = 2).

	fe	ti	ta_1_	ta_2_	ta_3_
P_1_	664–695, 680	469–503, 486	858–940, 899	455–489, 472	337–355, 346
P_2_	692–700, 696	604–638, 621	348–362, 355	171–189, 180	126–135, 131
P_3_	726–751, 739	783–801, 792	560–594, 577	291–302, 297	220–231, 226
	**ta_4_**	**ta_5_**	**LR**	**BV**	**SV**
P_1_	282–290, 286	147–152, 150	1.83–1.87, 1.85	1.62–1.67, 1.65	1.27–1.32, 1.30
P_2_	83–84, 84	75–78, 77	0.57–0.58, 0.57	3.50–3.61, 3.56	3.70–3.72, 3.71
P_3_	127–130, 129	90–93, 92	0.72–0.74, 0.73	2.82–2.86, 2.84	2.61–2.69, 2.65

***Hypopygium*** (Figs 4B, E, H, 6B, C). Tergite IX with triangular posterior margin, bearing 4–8, 6 setae at base of anal point. Laterosternite IX with two setae. Anal point originating from caudal margin of anal tergite, slightly wider at base, parallel-sided, swollen apically, 56–60, 58 μm long, 17–21, 19 μm wide at base, 10 μm wide at apex (Fig. 4E). Anal tergite bands V-shaped, fused in the middle. Phallapodeme 79–81, 80 μm long. Transverse sternapodeme 39–45, 42 μm long. Superior volsella wide, parallel-sided, bent inward at 1/3 distance from apex, apically rounded, 50–61, 56 μm long, 12–14, 13 μm wide at apex, bearing an apical seta and a proximal lateral seta, both arising from distinct setal pits, free microtrichia. Inferior volsella with distinct blunt caudal projection, not reaching beyond anal tergite margin, and covered by microtrichia (Fig. 4H). Gonocoxite 119–135, 127 μm long, with 4 strong medial setae. Gonostylus 171–174, 173 μm long, narrower at base, gradually widened distally, curved and parallel-sided, apically rounded, bearing 6–8, 7 setae along inner margin and one stronger seta at apex. HR 0.70–0.78, 0.74. HV 2.12–2.25, 2.18.

##### Etymology.

Name after Prof. Xin-Hua Wang, for his outstanding contribution towards increasing our knowledge of aquatic insect taxonomy; noun in nominative case.

##### Distribution.

China (Xizang).

#### ﻿ Parachironomus nankaiensis

Taxon classificationAnimaliaDipteraChironomidae

Liu & Lin sp. nov.

83E07836-0B81-5861-AFB2-0D88DD0C6484

https://zoobank.org/9524FD76-DA2D-4E37-90CD-EB6F4765A95D

Figs 3 , 4 , 7

##### Material examined.

***Holotype*.** Male (SHOU: XL599), China, Xizang Autonomous Region, Shannan City, Gongga County, Gangdui Town, 29.27888°N, 90.82323°E, 3586 m a.s.l., 18.VII.2014, sweep net, leg: X.L. Lin.

##### Diagnostic characters.

The species can be distinguished from known species of *Parachironomus* by the following combination of characters: frontal tubercles small; squama with seven setae; anal tergite bands V-shaped, separated; superior volsella narrower at base, curved and expanded in the distal part, with a bare lamellar projection as wide as apex of volsella; inferior volsella reaching slightly beyond anal tergite margin; gonostylus slender, slightly curved in the middle, tapered to the apex.

##### Description.

**Adult male** (*n* = 1). Total length 3.90 mm. Wing length 2.51 mm. Total length/wing length 1.56. Wing length/length of profemur 2.64.

***Coloration*.** Thorax dark brown with dark spots. Legs brown. Abdomen yellowish green to brown, tergites I–V yellowish green, tergites VI–VIII yellowish brown, hypopygium brown.

***Head*** (Fig. 3C). Antenna with 11 flagellomeres, ultimate flagellomere 699 μm long. AR 1.60. Frontal tubercles small, 9 μm long, 8 μm wide. Temporal setae 14, including 4 inner verticals; 4 outer verticals; and 6 postorbitals. Clypeus with 11 setae. Tentorium 154 μm long, 39 μm wide. Palpomere lengths (in μm): 30; 42; 115; 146; 235; Pm5/ Pm3 2.04.

***Thorax*** (Fig. 3F). Antepronotals with 3 setae, acrostichals 6, dorsocentrals 10, prealars 3. Scutellum with 10 setae.

**Figure 7.**
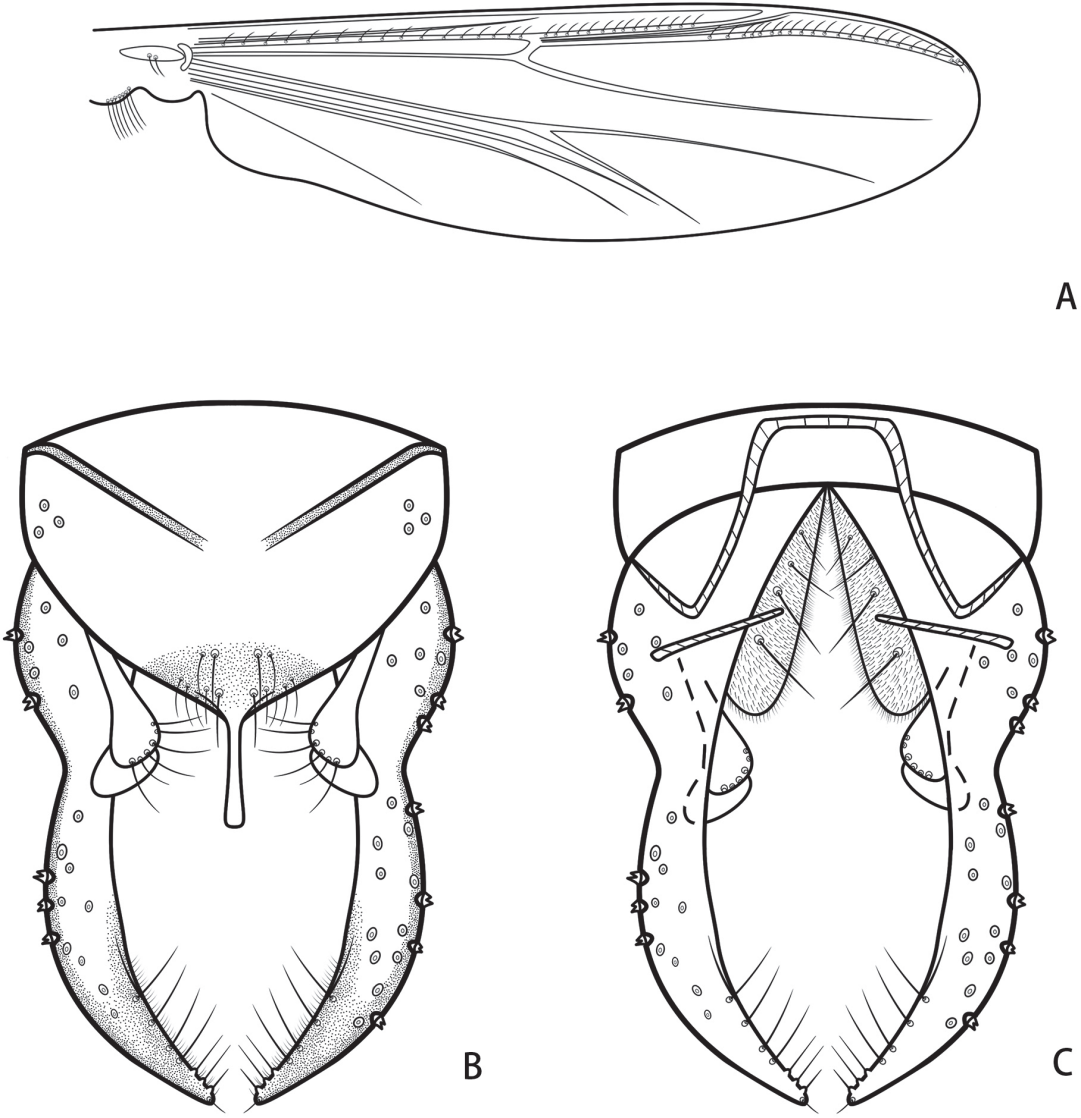
*Parachironomusnankaiensis* Liu & Lin, sp. nov. holotype male **A** wing **B** hypopygium, dorsal view **C** hypopygium, ventral view.

***Wing*** (Fig. 7A). VR 1.11. R with 16 setae, R_1_ with 14 setae, R_4+5_ with 22 setae. Brachiolum with two setae. Squama with seven setae.

***Legs*.** Front tibia with three subapical setae, 80, 87 and 93 μm long. Combs of mid tibia 33 μm wide with 19 μm long spur, and 29 μm wide with 27 μm long spur; combs of hind tibia 30 μm wide with 20 μm long spur, 65 μm wide with 36 μm long spur. Tarsomere 1 of mid and hind legs without sensilla chaetica. Lengths (in μm) and proportions of legs as in Table 5.

**Table 5.** Lengths (in μm) and proportions of legs of *Parachironomusnankaiensis* Liu & Lin, sp. nov., adult male (*n* = 1).

	fe	ti	ta_1_	ta_2_	ta_3_
P_1_	950	744	1135	576	431
P_2_	889	825	448	258	187
P_3_	1056	1072	675	385	292
	**ta_4_**	**ta_5_**	**LR**	**BV**	**SV**
P_1_	315	140	1.53	1.94	1.49
P_2_	120	91	0.54	3.30	3.83
P_3_	162	89	0.63	3.02	3.15

***Hypopygium*** (Figs 4C, F, I, 7B, C). Tergite IX with 14 setae at base of anal point and shoulder-like posterior margin of anal tergite. Laterosternite IX with three setae. Anal point originating from caudal margin of anal tergite, almost parallel-sided, moderately expanded at apex, 50 μm long, 9 μm wide at base, 10 μm wide apically. Anal tergite bands V-shaped, separated (Fig. 4F). Phallapodeme 80 μm long. Transverse sternapodeme 59 μm long. Superior volsella narrower at base, curved and expanded in the distal part, with a bare lamellar projection as wide as apex of volsella, 80 μm long, 22 μm wide at apex; and bearing six long setae at apex, free microtrichia. Inferior volsella with fairly blunt caudal projection, reaching slightly beyond anal tergite margin, and covered with microtrichia (Fig. 4I). Gonocoxite 143 μm long, with four stout setae placed along inner margin. Gonostylus 169 μm long, slender, slightly curved in the middle, tapered to the apex, bearing seven setae along inner margin and one single seta at apex. HR 0.85. HV 2.31.

##### Etymology.

Name after Nankai University, the institution of study and work for Prof. Xin-Hua Wang; noun in nominative case.

##### Distribution.

China (Xizang).

### ﻿Key to known adult males of *Parachironomus* from China

**Table:** 

1	Tergite IX with shoulder-like caudal margin	**2**
–	Tergite IX with triangle caudal margin	**4**
2	Superior volsella without projection; gonostylus with constriction in middle	***P.frequens* (Johannsen)**
–	Superior volsella with a projection; gonostylus gradually tapered to the top	**3**
3	AR 0.58–0.67; anal tergite bands fused; inferior volsella not reaching beyond anal tergite margin	***P.demissum* (Yan, Wang & Bu), comb. nov.**
–	AR 1.60; anal tergite bands separated; inferior volsella reaching slightly beyond anal tergite margin	***P.nankaiensis* Liu & Lin, sp. nov.**
4	Superior volsella short, bearing two apical setae and with folds on inner margin; gonostylus widened basally	***P.gracilior* (Kieffer)**
–	Superior volsella elongate, bearing an apical seta and a subapical seta and without folds; gonostylus widened distally	**5**
5	Mid and hind tibiae each with 1 spur; superior volsella straightly, widened at base	***P.poyangensis* Yan**
–	Mid and hind tibiae each with 2 spurs; superior volsella curved, widened in the distal part	**6**
6	Superior volsella slightly curved; gonostylus slender, with distinct expansion in distal 1/3	***P.monochromus* (van der Wulp)**
–	Superior volsella bent inward at1/3 distance from apex; gonostylus parallel-sided, gradually widened distally	***P.wangi* Liu & Lin, sp. nov.**

## ﻿Discussion

In this study, the holotype of *Paracladopelmademissum* were examined, and the original description has been modified. The distinguishing feature of *Parachironomus* are that the superior volsella usually has a distinct preapical tooth as well as setae arising from distinct pits (Yan et al. 2012; pers. comm. Martin SpiesJuly. 2022). We re-checked the holotype, and the characters of tergite IX with shoulder-like posterior margin, superior volsella slender and with 2 distal setae arising from distinct pits, inferior volsella with blunt or pointed caudal projection conform to the characters of *Parachironomus*; therefore, *Paracladopelmademissum* should be placed in *Parachironomus*. *Parachironomusdemissum* comb. nov. resembles *Parachironomusdigitalis* Edwards, 1929 in having similarly shaped tergite IX, anal point and superior volsella, but the antenna ratio and some other measurements are different.

*Parachironomuswangi* Liu & Lin, sp. nov. resembles *Parachironomusbiannulatus* Staeger, 1839 in having similar shapes of the superior volsella and posterior margin of tergite IX, but can be separated by the following combination characters: AR 1.54–1.55, anal point parallel-sided and gonostylus expanded apically in *P.wangi* Liu & Lin, sp. nov, whereas AR 3.0–3.6, the anal point is constricted in the middle, and the gonostylus is tapered to the apex in *P.biannulatus*.

*Parachironomusnankaiensis* Liu & Lin, sp. nov. resembles *Parachironomuscayapo* Spies, Fittkau & Reiss, 1994 in having similar shapes of anal point, inferior volsella, and anal tergite bands, but can be separated from the latter by the following combination characters: squama seven setae, the superior volsella expanded in the distal part and with a bare lamellar projection, plus with the gonostylus tapered to the apex. In contrast, the squama of *P.cayapo* is bare, the superior volsella is not widened in the distal part and has no projection, and the gonostylus has a protruding hump.

The results of molecular identification and morphological taxonomy are consistent, indicating that DNA barcodes and traditional morphological taxonomy are complementary in this case; therefore, the DNA barcode can be used as a simple method to supplement traditional morphological taxonomy for *Parachironomus*.

**Figure 8.**
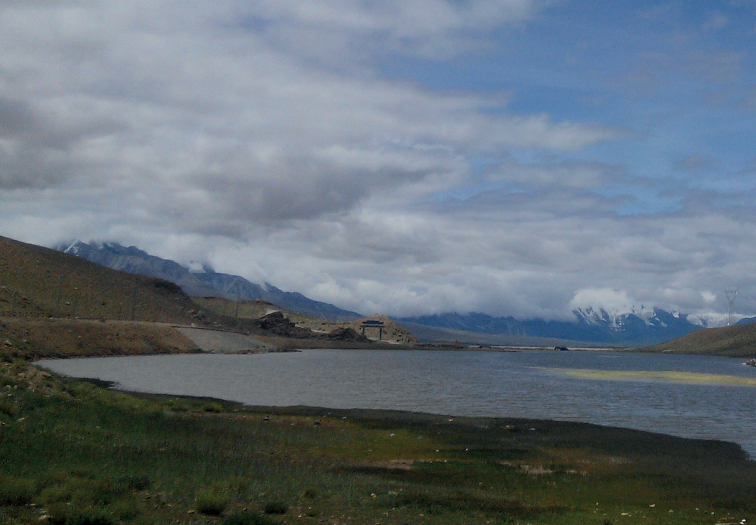
Habitat for *Parachironomuswangi*, Liu & Lin, sp. nov.

Biogeographically, the three species examined in this study are all distributed in the Tibetan Plateau at an altitude of more than 3,000 meters (Fig. 8). With the gradual increase of altitude, the climate gradually deteriorates, and *Parachironomus* living in high altitude areas have strong cold tolerance. The high biodiversity in Tibetan Plateau is demonstrated, as well as the distribution range of this genus extended.

In conclusion, this study not only enriches the database of Chironomidae in China, but also provides baseline data for the protection of the environment and biodiversity in the Tibetan Plateau.

## Supplementary Material

XML Treatment for Parachironomus demissum

XML Treatment for Parachironomus wangi

XML Treatment for Parachironomus nankaiensis
